# Triglyceride–Glucose Index Independently Predicts New-Onset Atrial Fibrillation After Septal Myectomy for Hypertrophic Obstructive Cardiomyopathy Beyond the Traditional Risk Factors

**DOI:** 10.3389/fcvm.2021.692511

**Published:** 2021-07-23

**Authors:** Zhipeng Wei, Enjun Zhu, Changwei Ren, Jiang Dai, Jinhua Li, Yongqiang Lai

**Affiliations:** Department of Cardiac Surgery, Institute of Heart, Lung and Blood Vascular Diseases, Beijing Anzhen Hospital, Capital Medical University, Beijing, China

**Keywords:** insulin resistance, TyG index, hypertrophic cardiomyopathy, postoperative atrial fibrillation, septal myectomy

## Abstract

The triglyceride–glucose index is a valuable marker of insulin resistance. However, the predictive value of this index for postoperative atrial fibrillation in patients undergoing septal myectomy remains unclear. A total of 409 patients with hypertrophic obstructive cardiomyopathy who underwent septal myectomy were recruited in this study. The triglyceride–glucose index was calculated for all patients preoperatively. All patients underwent clinical data collection, blood sampling, and standard echocardiographic examinations during hospitalization. The prevalence of postoperative atrial fibrillation was approximately 15% in the present study. Multivariate logistic regression revealed that age (odds ratio [OR]: 1.053, 95% CI: 1.016–1.090, *P* = 0.004), hypertension (OR: 2.399, 95% CI: 1.228–4.686, *P* = 0.010), left atrial diameter (OR: 1.101, 95% CI: 1.050–1.155, *P* < 0.001), and triglyceride–glucose index (OR: 4.218, 95% CI: 2.381–7.473, *P* < 0.001) were independent risk factors for postoperative atrial fibrillation in patients undergoing septal myectomy. In receiver operating characteristic curve analysis, the triglyceride–glucose index could provide a moderate predictive value for postoperative atrial fibrillation after septal myectomy 0.723 (95% CI: 0.650–0.796, *P* < 0.001). Moreover, adding the triglyceride–glucose index to conventional risk factor model could numerically but not significantly increase our ability to predict postoperative atrial fibrillation (area under the receiver: 0.742 (0.671–0.814) vs. 0.793 (0.726–0.860), *p* = 0.065) after septal myectomy. In our retrospective cohort study, the triglyceride–glucose index was identified as an independent predictor of postoperative atrial fibrillation in patients undergoing septal myectomy.

## Introduction

Postoperative atrial fibrillation (POAF) has a prevalence of up to 24.6% in patients with hypertrophic obstructive cardiomyopathy (HOCM) who undergo septal myectomy (1). It is associated with increased morbidity and mortality as well as prolonged intensive care unit and hospital stay (2, 3). Several risk factors, including age, male sex, valvular surgery, and left atrial enlargement, have been reported to be associated with POAF in patients who underwent cardiac surgery (4, 5); however, no effective tool based on clinical parameters has been developed to predict POAF to date. It is crucial to identify patients at high risk of developing POAF before surgery so that modifiable risk factors can be addressed. Additionally, further determination of such factors is essential.

Insulin resistance (IR) is a known risk factor for cardiovascular disease (6) that is observed in patients with hypertrophic cardiomyopathy (7). It is associated with atrial remodeling (8) as well as atrial fibrillation (AF) (9). IR may predispose to AF by increasing left atrial size or impairing left ventricular diastolic function in hypertrophic cardiomyopathy (10); however, its role in predicting new-onset POAF after septal myectomy in these patients has not been well investigated.

The hyperinsulinemic–euglycemic clamp, which is considered the “gold standard” test for IR assessment, can accurately assess the severity of IR by measuring the glucose metabolism rate and the sensitivity of an individual's peripheral tissues to insulin (11). However, it is not commonly used in the clinical setting because of the complexity of the test and the associated costs. The more commonly used homeostasis model assessment of IR method requires measurement of fasting insulin levels, which is not routinely done. Therefore, the triglyceride–glucose (TyG) index, which combines triglyceride and fasting plasma glucose levels, has been proposed alternatively. According to previous studies, the TyG index can assess IR, more accurately than homeostasis model assessment of IR (12, 13). This study aimed to assess the impact of preoperative TyG levels on new-onset POAF in patients with HOCM undergoing septal myectomy.

## Methods

A total of 409 patients without a history of preoperative atrial arrhythmia, who underwent septal myectomy in Beijing Anzhen Hospital between September 2009 and June 2020 were enrolled in this study. The diagnostic criteria for HOCM include unexplained septal hypertrophy with a septal thickness of >15 mm and left ventricular outflow tract gradient (LVOTG) of ≥30 mmHg, according to previous guidelines (14). Septal myectomy was performed if patients had severe symptoms despite optimal medical therapy and LVOTG of ≥50 mmHg at rest or with exertion. The surgical method is described as follows: septal myectomy was performed through transverse aortotomy extending into the non-coronary sinus. Continuous resection was commenced at the nadir of the right aortic sinus leftward toward the mitral annulus and apically to the base of the papillary muscles. All areas of papillary muscle fusion to the septum or ventricular free wall were divided, and anomalous chordal structures and fibrous attachments of the mitral leaflets to the ventricular septum or free wall were divided or excised (15). Patients with conditions that could affect IR or the TyG index, such as severe liver, kidney, and thyroid disease were excluded. The study was approved by the ethics committee of Beijing Anzhen Hospital, Capital Medical University and conducted in accordance with the Declaration of Helsinki. Written informed consent was obtained from all participants.

The demographics and clinical characteristics of each patient were recorded. All patients underwent routine laboratory tests (after a fasting period of 12 h) on admission. Fasting serum triglyceride and glucose levels were measured using an automated biochemistry analyzer (Roche Cobas 801, Germany). The TyG index was calculated using this formula: ln [fasting TG (mg/dL) × fasting plasma glucose (mg/dL)/2]. Data were collected by two independent researchers.

Patients underwent continuous electrocardiographic monitoring or a standard 12-lead routine electrocardiograph daily during the postoperative period after septal myectomy and until discharge. POAF was defined as the presence of AF that lasted ≥5 min or required cardioversion with antiarrhythmic drugs.

Echocardiographic studies were performed using a commercially available E9 ultrasound system. Left atrial diameter (LAD), left ventricular end-diastolic dimension, left ventricular ejection fraction, maximal wall thickness, and LVOTG were measured. Values were analyzed according to the American Society of Echocardiography guidelines (16).

Continuous data are presented as median and interquartile range or mean ± standard deviation. Categorical variables are presented as number and percentage. Intergroup comparisons of normally distributed numerical variables were performed using an independent sample *t*-test. The Mann–Whitney U test was performed for intergroup comparisons of non-normally distributed numerical variables. Categorical variables were compared using the chi-square test. The cut-off value for the TyG index was determined by the area under the receiver operating characteristic (ROC) curve. Multivariate logistic regression models were used to determine the risk factors for POAF. Variables with a *P*-value of <0.1, on univariate analysis, were entered into the multivariate analysis. Co-linearity and intercorrelations among variables were also taken into consideration when constructing the multivariate logistic regression analysis model to control for confounding variables. Finally, the c-statistic of the conventional risk factor model vs. the conventional risk factor plus TyG index model were compared using the Delong test to examine whether adding the TyG index to conventional risk factors could improve discrimination power for POAF. All reported probability values were two-tailed, and a *P*-value of <0.05 was considered statistically significant. SPSS version 26.0 (IBM) and MedCalc were used for data analysis.

## Results

As presented in Table 1, 409 patients who underwent septal myectomy were included. The enrolled patients were followed up for a median of 8 days (6–11). POAF was diagnosed in 61 (15%) patients during their postoperative course. Compared with patients without POAF, those with POAF were older (49.92 ± 12.35 vs. 56.75 ± 10.85 years, *P* < 0.001), had higher body mass index (25.08 ± 3.33 vs. 26.41 ± 3.22, *P* < 0.004), and had longer mechanical ventilation time (16.92 [13.02–21.56] vs. 18.92 [16.04–38.42] h, *P* < 0.001) and postoperative hospital stay (8 [6–11] vs. 9 [7–12.50] days, *P* = 0.028). Patients in the POAF group were more likely to have hypertension (21.3% vs. 49.2%, *P* < 0.001), diabetes (7.2% vs. 23.0%, *P* < 0.001), hyperlipidemia (12.1% vs. 31.1%, *P* < 0.001), and elevated serum glucose (*P* = 0.002), total cholesterol (*P* < 0.001), triglycerides (*P* < 0.001), and low-density lipoprotein cholesterol (*P* = 0.004) levels. The TyG index was significantly higher (6.90 ± 0.55 vs. 7.41 ± 0.67, *P* < 0.001) in the POAF group. The echocardiographic data are summarized in Table 2. Patients with POAF had larger LAD both preoperatively (42.31 ± 6.27 vs. 5.10 ± 7.70 mm, *P* = 0.003) and postoperatively (37.00 ± 6.17 vs. 39.66 ± 6.57 mm, *P* = 0.004). There were no significant associations between POAF and other echocardiographic variables.

**Table 1:** Perioperative clinical variables of patients.

**Variables**	**Whole cohort (*n* = 409)**	**No POAF (*n* = 348)**	**POAF (*n* = 61)**	***P-value***
**Preoperative data**				
Age (years)	50.94 ± 12.37	49.92 ± 12.35	56.75 ± 10.85	<0.001
Male (%)	213 (52.1)	181 (52.0)	32 (52.5)	0.949
Hypertension (%)	104 (25.4)	74 (21.3)	30 (49.2)	<0.001
Diabetes mellitus (%)	39 (9.5)	25 (7.2)	14 (23.0)	<0.001
Hyperlipemia (%)	61 (14.9)	42 (12.1)	19 (31.1)	<0.001
CAD (%)	59 (14.4)	47 (13.5)	12 (19.7)	0.206
Body mass index (kg/m^2^)	25.28 ± 3.34	25.08 ± 3.33	26.41 ± 3.22	0.004
**Medication**				
Calcium channel blockers (%)	270 (66.0)	231 (66.4)	39 (63.9)	0.710
Beta-blockers (%)	291 (71.1)	245 (70.4)	46 (75.4)	0.426
**Laboratory factors**				
Glucose (mmol/L)	5.32 ± 1.48	5.17 ± 1.19	6.21 ± 2.42	0.002
ALT (IU/L)	19.00 (14.00–29.00)	19.00 (14.00–29)	19.50 (15.00–32.75)	0.482
AST (IU/L)	21.00 (18.00–27.00)	21.00 (18.00–26.50)	21.50 (18.00–28.00)	0.630
Creatinine (μmol)	68.95 (59.30–79.68)	68.45 (58.93–79.00)	72.95 (62.53–89.00)	0.068
TC (mmol/L)	4.49 ± 0.89	4.41 ± 0.86	4.92 ± 0.95	<0.001
HDL-C (mmol/L)	1.12 ± 0.25	1.12 ± 0.24	1.09 ± 0.28	0.400
LDL-C (mmol/L)	2.81 ± 0.78	2.76 ± 0.76	3.08 ± 0.84	0.004
TG (mmol/L)	1.47 ± 0.88	1.38 ± 0.79	1.99 ±1.16	<0.001
TyG index	6.97 ± 0.60	6.90 ± 0.55	7.41 ± 0.67	<0.001
**Concomitant operative procedures**				
Coronary artery bypass grafting (%)	40 (9.8)	31 (8.9)	12 (14.8)	0.156
Myocardial unroofing (%)	16 (3.9)	13 (3.7)	3 (4.9)	0.935
Mitral valve replacement (%)	87 (21.3)	71 (20.4)	16 (26.2)	0.305
Mitral valve repair (%)	67 (16.4)	59 (17.0)	8 (13.1)	0.455
Aortic valve replacement or repair (%)	25 (6.1)	20 (5.7)	5 (8.2)	0.655
Tricuspid valve replacement or repair (%)	28 (6.8)	23 (6.6)	5 (8.2)	0.859
**Postoperative data**				
Mechanical ventilation time (hours)	17.25 (13.46–22.00)	16.92 (13.02–21.56)	18.92 (16.04–38.42)	<0.001
Aortic clamp time (minutes)	74.00 (55.50–102.00)	73.50 (54.00–101.00)	75.00 (58.50–117.50)	0.133
operation time (h)	4.25 (3.58–5.17)	4.25 (3.58–5.17)	4.25 (3.88–5.30)	0.396
cardiopulmonary bypass time (minutes)	114.00 (88.00–149.00)	113.00 (86.00–148.75)	118.00 (98.50–153.50)	0.126
Post-operative hospital stays (days)	8.00 (6.00–11.00)	8.00 (6.00–11.00)	9.00 (7.00–12.50)	0.028

*POAF, postoperative atrial fibrillation; CAD, coronary artery disease; ALT, alanine aminotransferase; AST, aspartate aminotransferase; TC, total cholesterol; HDL-C, high density lipoprotein cholesterol; LDL-C, low density lipoprotein cholesterol; TG, triglyceride; TyG index, triglyceride glucose index*.

**Table 2:** Perioperative echocardiographic variables of patients.

**Variables**	**Whole cohort (*n* = 409)**	**No POAF (*n* = 348)**	**POAF (*n* = 61)**	***P-value***
**Preoperative data**				
Maximum wall thickness (mm)	21.14 ± 5.28	21.31 ± 5.35	20.21 ± 4.83	0.143
Left atrial diameter (mm)	42.75 ± 6.58	42.31 ± 6.27	45.10 ± 7.70	0.003
Left ventricular end-diastolic diameter (mm)	43.65 ± 5.29	43.63 ± 5.21	43.74 ± 5.75	0.886
Left ventricular ejection fraction (%)	67.36 ± 6.39	67.41 ± 6.34	67.07 ± 6.73	0.710
Peak LVOT gradients (mmHg)	84.00 (60.00–106.00)	83.00 (57.75–106.00)	85.00 (73.00–109.00)	0.120
Moderate or severe MR (%)	252 (61.6)	210 (60.3)	42 (68.9)	0.208
**Postoperative data**				
Left atrial diameter (mm)	37.40 ± 6.30	37.00 ± 6.17	39.66 ± 6.57	0.004
Left ventricular end-diastolic diameter (mm)	42.25 ± 5.31	42.14 ± 5.32	42.86 ± 5.23	0.342
Left ventricular ejection fraction (%)	60.87 ± 7.67	61.20 ± 7.61	59.09 ± 7.85	0.053
Peak LVOT gradients (mmHg)	18.00 (12.00–25.50)	18.00 (12.00–25.00)	20.50 (12.00–37.50)	0.281
Moderate or severe MR (%)	15 (3.7)	12 (3.4)	3 (4.9)	0.846

*POAF, postoperative atrial fibrillation; LVOT, left ventricular outflow tract; MR, mitral regurgitation. Peak LVOT gradients was defined as the maximal value at resting or after provocation*.

Patients were divided into the “low TyG index group” (<7.60) and “high TyG index group” (≥7.60) according to the cut-off point which is identified by ROC curve analysis. The high TyG index group (*n* = 60) had higher rates of hypertension (41.7% vs. 22.6%, *P* = 0.002), diabetes (31.7% vs. 5.7%, *P* < 0.001), hyperlipidemia (46.7% vs. 9.5%, *P* < 0.001), and coronary artery disease (25.0% vs. 12.6%, *P* = 0.012), as well as higher serum glucose (*P* < 0.001), total cholesterol (*P* < 0.001), and triglyceride (*P* < 0.001) levels. In addition, POAF occurred more frequently in the high TyG index group (45.0% vs. 9.7%, *P* < 0.001) (Details shown in Supplementary Table 1).

Univariate analysis showed that preoperative variables including age (odds ratio [OR]: 1.054, 95% CI: 1.027–1.082, *P* < 0.001), body mass index (OR: 1.126, 95% CI: 1.037–1.223, *P* = 0.005), hypertension (OR: 3.583, 95% CI: 2.039–6.297, *P* < 0.001), diabetes mellitus (OR: 3.849, 95% CI: 1.869–7.924, *P* < 0.001), hyperlipemia (OR: 3.296, 95% CI: 1.754–6.192, *P* < 0.001), LAD (OR: 1.063, 95% CI: 1.020–1.108, *P* = 0.004), and TyG index (OR: 4.163, 95% CI: 2.551–6.794, *P* < 0.001) were risk factors for the occurrence of POAF, whereas male sex, left ventricular ejection fraction, maximum wall thickness, LVOTG, and moderate or severe mitral regurgitation were not. In the multivariate analysis, age (OR: 1.053, 95% CI: 1.016–1.090, *P* = 0.004), hypertension (OR: 2.399, 95% CI: 1.228–4.686, *P* = 0.010), LAD (OR: 1.101, 95% CI: 1.050–1.155, *P* < 0.001), and TyG index (OR: 4.218, 95% CI: 2.381–7.473, *P* < 0.001) were independent risk factors for POAF in patients undergoing septal myectomy (Table 3).

**Table 3:** Logistic analysis for predictors of postoperative atrial fibrillation.

**Characteristics**	**OR**	**95% CI**	***P-value***
**Univariate Logistic regression analysis**			
Age	1.054	1.027–1.082	<0.001
Male	0.982	0.570–1.694	0.949
Body mass index	1.126	1.037–1.223	0.005
Hypertension	3.583	2.039–6.297	<0.001
Diabetes mellitus	3.849	1.869–7.924	<0.001
Hyperlipemia	3.296	1.754–6.192	<0.001
Left atrial diameter	1.063	1.020–1.108	0.004
Left ventricular ejection fraction	0.992	0.949–1.036	0.709
Maximum wall thickness	0.958	0.905–1.015	0.144
LVOT gradients	1.004	0.997–1.010	0.319
Moderate or severe mitral regurgitation	1.453	0.811–2.602	0.209
TyG index	4.163	2.551–6.794	<0.001
**Multivariate Logistic regression analysis^a^**			
Age	1.053	1.016–1.090	0.004
Hypertension	2.399	1.228–4.686	0.010
Left atrial diameter	1.101	1.050–1.155	<0.001
TyG index	4.218	2.381–7.473	<0.001

*OR, odds ratio; CI, confidence interval; LVOT, left ventricular outflow tract; TyG index, triglyceride glucose index*.

a *Age, Male, Body mass index, Hypertension, Diabetes mellitus, Hyperlipemia, Left atrial diameter, and TyG index were included in the multivariate logistic regression analysis*.

**Figure 1:**
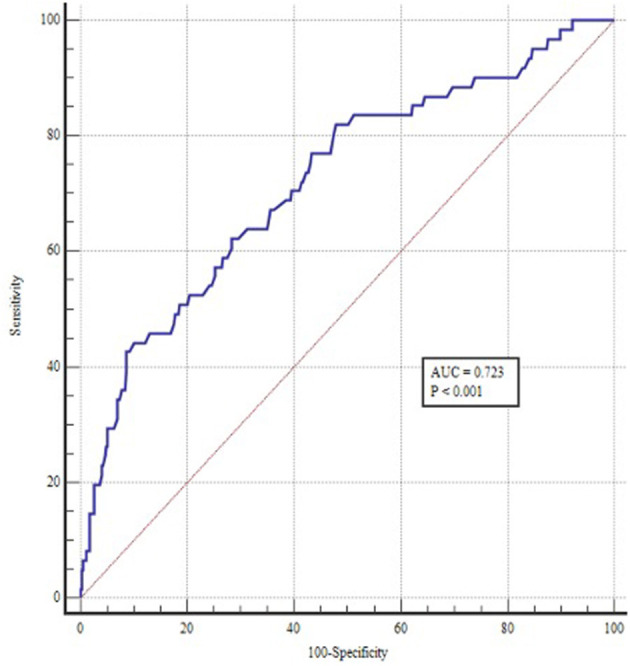
ROC curve of TyG index for predicting POAF in patients undergoing septal myectomy. The optimal cut-off point was 7.60 with sensitivity and specificity of 44.3 and 90.5% (area under the curve 0.723, 95% CI: 0.650 to 0.796, *P* < 0.001).

**Figure 2:**
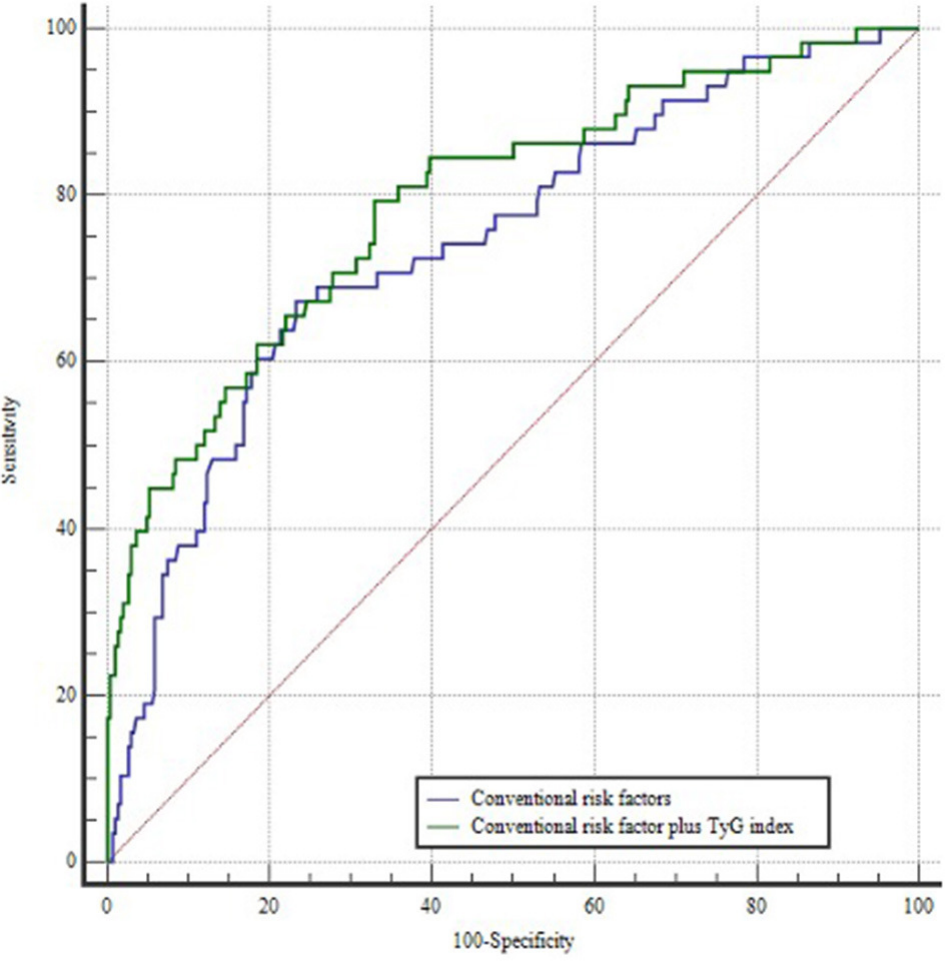
ROC curves of the conventional risk factor model and the conventional risk factor plus TyG index model. The conventional risk factor model includes age, hypertension, and left atrial diameter.

A ROC curve was constructed to assess the ability of the TyG index to identify patients at risk of POAF (Figure 1). The optimal value for predicting POAF after septal myectomy was 7.60, with a sensitivity of 44.3% and specificity of 90.5%. The area under the receiver curve for TyG index was 0.723 (95% CI: 0.650–0.796, *P* < 0.001). Moreover, as shown in Figure 2, the TyG index could numerically but not significantly increase the ability of the conventional risk factor model to predict the incidence of POAF in patients after septal myectomy (area under the receiver curve: 0.742 (0.671–0.814) vs. 0.793(0.726–0.860), *P* = 0.065).

## Discussion

The predictive value of IR for new-onset POAF in patients who undergo septal myectomy for HOCM has not been adequately studied. In the present study, we used the TyG index as a biomarker of IR to answer this question. The main finding of our study is that the TyG index is an independent predictor of POAF in patients who underwent septal myectomy beyond the traditional risk factors. Additionally, our present study suggested that the TyG index could provide moderate predictive value for POAF in patients who underwent septal myectomy. And adding the TyG index to conventional risk factor model could numerically but not significantly increase our ability to predict POAF after septal myectomy. To the best of our knowledge, this is the first study to capture the value of the TyG index for predicting new-onset POAF in patients who underwent septal myectomy.

POAF is a common complication of cardiac surgery (5, 17). In this study, the prevalence of POAF was approximately 15%, which is lower than the previously reported 20% in patients undergoing septal myectomy (18). The lower incidence of POAF in our study is partly because we excluded patients with preoperative AF. It has been reported that patients with POAF after coronary revascularization or valvular surgery have increased postoperative morbidity and mortality as well as prolonged intensive care unit and hospital stay (2, 19). Consistent with these studies, we also found that patients with POAF have longer mechanical ventilation time and postoperative hospital stay than those without. Long-term outcomes in inpatients with POAF are worse than in those with sinus rhythm, but similar to those with preoperative AF (3, 20, 21). Considering the important effects of POAF on short- and long-term clinical outcomes, it is necessary to identify its risk factors in patients undergoing septal myectomy. Some of these risk factors, including advanced age, hypertension, and LAD, have been previously identified (3, 22).

The TyG index has been demonstrated as a useful predictor of cardiovascular events (23, 24). It has been proposed to be a reproducible, reliable, and valid surrogate marker of IR because it is simpler and more cost-effective than the gold standard hyperinsulinemic–euglycemic clamp (25–27). In this study, we used the TyG index to further investigate the relationship between IR and POAF in patients undergoing septal myectomy. The results showed that patients with POAF had a higher TyG index than those without POAF.

The TyG index had a moderate predictive value for POAF in patients who underwent septal myectomy. The relationship between TyG index and POAF was still significant even after adjusting for sex, body mass index, and other variables. When we divided patients according to the optimal TyG value for predicting POAF, we found that the TyG index was associated with common cardiovascular comorbidities. Adding the TyG index to the conventional risk factors model could numerically but not significantly increase our ability to predict POAF in patients who underwent septal myectomy due to HOCM.

However, patients after septal myectomy are continuously monitored using telemetry, which is much faster and more accurate in detecting AF. Our present study is significant for clinical practice to optimize the management of patients who underwent septal myectomy for the following reasons. First, we may identify patients at high risk of POAF preoperatively using the TyG index. Then, we may give those patients at high risk of POAF the intensive pre-treatment such as statin, which may reduce the risk of POAF according to previous study (1). Additionally, our present study indicated that the TyG index could serve as an independent risk factor for the incidence of POAF after septal myectomy beyond the traditional risk factors. From the translational outlook, this finding may improve our understanding of the pathogenesis of POAF and open new avenues for the primary prevention of POAF. However, before using it in clinical practice, we should further check the discrimination power of TyG in the validation cohort.

The exact mechanism by which TyG index is closely related to POAF is still unclear. However, we speculate that TyG is a reliable marker of IR, which may be mainly based on the intrinsic association. IR refers to a clinical condition in which the biological effect of insulin is lower than expected. It can lead to metabolic syndrome and associated organ dysfunction (28). IR is a possible mechanism for the occurrence of AF and commonly occurs in patients with hypertrophic cardiomyopathy (7, 10). IR can induce atrial electrical and structural remodeling through activation of the MAPK pathway (8) and disrupt the functionality of insulin-sensitive glucose transporters, thus predisposing to AF (29). In addition, IR may lead to AF by increasing the left atrial size and left ventricular diastolic dysfunction (10).

The TyG index is a readily available and convenient IR biomarker, which, as our study shows, can be used for the identification of individuals at high risk of POAF among patients undergoing septal myectomy and can be used in conjunction with other established risk factors for this purpose.

Our study has some limitations. First, due to the inherent limitation of retrospective study, not all potential confounders affecting IR and /or POAF, such as lifestyle, dietary habits, and area of septal myectomy, were determined. Second, we only measured fasting plasma triglyceride and glucose levels once on admission; thus, we could not determine the effect of dynamic changes of the TyG index on POAF. Third, we do not have long-term follow-up information on the impact of the TyG index on POAF. Fourth, we failed to determine the discriminative power of TyG in the validation cohort due to the relatively small sample size; therefore, future studies are required to confirm our findings. Finally, we only enrolled patients with HOCM who underwent septal myectomy, which may limit the generalizability of our findings to other cardiac surgery populations.

In conclusion, we identified TyG index as an independent risk factor for POAF in patients undergoing septal myectomy in our retrospective cohort study. However, before putting it into clinical practice, validation of these results by large prospective, multicenter trials is needed.

## Data Availability Statement

The original contributions presented in the study are included in the article/Supplementary Material, further inquiries can be directed to the corresponding author/s.

## Ethics Statement

The studies involving human participants were reviewed and approved by Medical ethics committee, Beijing Anzhen Hospital, Capital Medical University. The patients/participants provided their written informed consent to participate in this study.

## Author Contributions

ZW: conceptualization, methodology, and writing—original draft preparation. EZ: data curation and software. CR: visualization and investigation. JD: supervision. JL: software and validation. YL: writing—reviewing and editing. All authors contributed to the article and approved the submitted version.

## Conflict of Interest

The authors declare that the research was conducted in the absence of any commercial or financial relationships that could be construed as a potential conflict of interest.

## Publisher's Note

All claims expressed in this article are solely those of the authors and do not necessarily represent those of their affiliated organizations, or those of the publisher, the editors and the reviewers. Any product that may be evaluated in this article, or claim that may be made by its manufacturer, is not guaranteed or endorsed by the publisher.

## Abbreviations

AF: Atrial fibrillation
IR: Insulin resistance
LAD: Left atrial diameter
LVOTG: Left ventricular outflow tract gradient
POAF: Postoperative atrial fibrillation
ROC: Receiver operating characteristic.
