# Supplementary material for: Triglyceride–Glucose Index Independently Predicts New-Onset Atrial Fibrillation After Septal Myectomy for Hypertrophic Obstructive Cardiomyopathy Beyond the Traditional Risk Factors
Source: Front Cardiovasc Med. 2021 Jul 23;8:692511. doi: 10.3389/fcvm.2021.692511 (PMC8342798; doi:10.3389/fcvm.2021.692511)
Supplement: Supplementary file 1 [file Table_1.DOCX]

Supplementary Material

# Supplementary Table

| **Supplementary Table 1 Comparison of clinical variables between low and high TyG index groups** | | | |
| --- | --- | --- | --- |
| **Characteristics** | **Low TyG index group (n=349)** | **High TyG index group (n=60)** | ***P-value*** |
| **Preoperative data** |  |  |  |
| Age (years) | 50.68±12.59 | 52.43±10.95 | 0.312 |
| Male (%) | 177 (50.7) | 36 (60.0) | 0.184 |
| Body mass index (kg/m^2^) | 25.17±3.38 | 25.92±3.07 | 0.106 |
| Hypertension (%) | 79 (22.6) | 25 (41.7) | **0.002** |
| Diabetes mellitus (%) | 20 (5.7) | 19 (31.7) | **<0.001** |
| Hyperlipemia (%) | 33 (9.5) | 28 (46.7) | **<0.001** |
| CAD (%) | 44 (12.6) | 15 (25.0) | **0.012** |
| POAF (%) | 34 (9.7) | 27 (45.0) | **<0.001** |
| **Echocardiographic variables** |  |  |  |
| Left atrial diameter (mm) | 43.01±6.69 | 41.17±5.70 | 0.063 |
| LV end-diastolic diameter (mm) | 43.64±5.20 | 43.69±5.85 | 0.950 |
| LV ejection fraction (%) | 67.36±6.35 | 67.31±6.69 | 0.953 |
| Maximum wall thickness (mm) | 21.31±5.27 | 20.09±5.27 | 0.123 |
| LVOT gradients (mmHg) | 85.00 (60.25-107.75) | 78.00 (54.50-97.00) | 0.191 |
| Moderate or severe MR (%) | 214 (61.3) | 38 (63.3) | 0.767 |
| **Laboratory factors** |  |  |  |
| Glucose (mmol/L) | 5.04±0.73 | 6.94±2.98 | **<0.001** |
| ALT (IU/L) | 19.00 (14.00-29.00) | 20.00 (16.00-30.00) | 0.142 |
| AST (IU/L) | 21.00 (18.00-27.00) | 21.00 (18.00-26.00) | 0.864 |
| Creatinine (μmol) | 68.00 (58.90-78.30) | 75.10 (64.50-91.00) | **0.001** |
| TC (mmol/L) | 4.41±0.84 | 4.97±1.00 | **<0.001** |
| HDL-C (mmol/L) | 1.14±0.25 | 1.01±0.23 | **<0.001** |
| LDL-C (mmol/L) | 2.79±0.76 | 2.91±0.88 | 0.268 |
| TG (mmol/L) | 1.22±0.50 | 2.96±1.17 | **<0.001** |
| TyG index | 6.80±0.44 | 7.98±0.37 | **<0.001** |
| **TyG index, triglyceride glucose index; CAD, coronary artery disease; POAF, postoperative atrial fibrillation; LV, left ventricular; LVOT, left ventricular outflow tract; ALT, alanine aminotransferase; AST, aspartate aminotransferase; TC, total cholesterol; HDL-C, high density lipoprotein cholesterol; LDL-C, low density lipoprotein cholesterol; TG, triglyceride.** | | | |
